# Comparison of the prevalence of respiratory viruses in patients with acute respiratory infections at different hospital settings in North China, 2012–2015

**DOI:** 10.1186/s12879-018-2982-3

**Published:** 2018-02-08

**Authors:** Jianxing Yu, Zhengde Xie, Tiegang Zhang, Yanqin Lu, Hongwei Fan, Donghong Yang, Thomas Bénet, Philippe Vanhems, Kunling Shen, Fang Huang, Jinxiang Han, Taisheng Li, Zhancheng Gao, Lili Ren, Jianwei Wang

**Affiliations:** 1 MOH Key Laboratory of Systems Biology of Pathogens and Christophe Mérieux Laboratory, IPB, CAMS-Fondation Mérieux, Institute of Pathogen Biology (IPB), Chinese Academy of Medical Sciences (CAMS) & Peking Union Medical College, Beijing, 100730 People’s Republic of China; 2 Key Laboratory of Major Diseases in Children and National Key Discipline of Pediatrics (Capital Medical University), Ministry of Education, Beijing Pediatric Research Institute, Beijing Children’s Hospital, Capital Medical University, No. 56 Nan-li-shi Road, Beijing, 100045 People’s Republic of China; 3 Beijing Center for Disease Control and Prevention, No.16, Hepingli Middle Avenue of Dongcheng district, Beijing, 100013 People’s Republic of China; 4 Shandong Medicinal Biotechnology Centre, Key Laboratory for Modern Medicine and Technology of Shandong Province, Shandong Academy of Medical Sciences, No. 18877 Jingshi Road, Jinan, Shandong 250062 People’s Republic of China; 5 Peking Union Medical College Hospital, Chinese Academy of Medical Sciences & Peking Union Medical College, Beijing, 100730 People’s Republic of China; 6 Department of Respiratory and Critical Care Medicine, Peking University People’s Hospital, Beijing, 100044 People’s Republic of China; 7 Service d’Hygiène, Epidémiologie et Prévention, Hôpital Edouard Herriot, Lyon, France; 8 Laboratoire des Pathogènes Emergents - Fondation Mérieux, Centre International de Recherche en Infectiologie (CIRI), Institut National de la Santé et de la Recherche Médicale U1111, Centre National de la Recherche Scientifique, UMR5308, Ecole Normale Supérieure de Lyon, Université Claude Bernard Lyon 1, 21, Avenue Tony Garnier, 69007 Lyon, France; 9 INSERM, F-CRIN, I-REIVAC, Lyon Collaborative Center, Lyon, France

**Keywords:** Respiratory tract infections, Viruses, Risk factors, Hospitalization, Sentinel surveillance

## Abstract

**Background:**

Acute respiratory infections (ARIs) are a great public health challenge globally. The prevalence of respiratory viruses in patients with ARIs attending at different hospital settings is fully undetermined.

**Methods:**

Laboratory-based surveillance for ARIs was conducted at inpatient and outpatient settings of 11 hospitals in North China. The first 2–5 patients with ARIs were recruited in each hospital weekly from 2012 through 2015. The presence of respiratory viruses was screened by PCR assays. The prevalence of respiratory viruses was determined and compared between patients at different hospital settings.

**Results:**

A total of 3487 hospitalized cases and 6437 outpatients/Emergency Department (ED) patients were enrolled. The most commonly detected viruses in the hospitalized cases were respiratory syncytial virus (RSV, 33.3%) in children less than two years old, adenoviruses (13.0%) in patients 15–34 years old, and influenza viruses (IFVs, 9.6%) in patients ≥65 years. IFVs were the most common virus in outpatient/ED patients across all age groups (22.7%). After controlling for the confounders caused by other viruses and covariates, adenoviruses (adjusted odds ratio [aOR]: 3.97, 99% confidence interval [99% CI]: 2.19–7.20) and RSV (aOR: 2.04, 99% CI: 1.34–3.11) were independently associated with increased hospitalization in children, as well as adenoviruses in adults (aOR: 2.14, 99% CI: 1.19–3.85). Additionally, co-infection of RSV with IFVs was associated with increased hospitalization in children (aOR: 12.20, 99% CI: 2.65–56.18).

**Conclusions:**

A substantial proportion of ARIs was associated with respiratory viruses in North China. RSV, adenoviruses, and co-infection of RSV and IFVs were more frequent in hospitalized children (or adenoviruses in adults), which might predict the severity of ARIs. Attending clinicians should be more vigilant of these infections.

**Electronic supplementary material:**

The online version of this article (10.1186/s12879-018-2982-3) contains supplementary material, which is available to authorized users.

## Background

Acute respiratory infections (ARIs) are a major global public health problem because of their high morbidity and mortality [1]. They represented 18.8 billion upper respiratory tract infections (URTIs), 150 million lower respiratory tract infections (LRTIs) and 2.6 million deaths in the global burden of diseases estimates in 2013 [2, 3]. In a substantial proportion of patients, ARIs are found to be associated with respiratory viruses (RVs) [1, 4–6]. In addition to their high frequency, infection with RVs can lead to severe outcomes, including hospitalization and death [1]. Notably, respiratory syncytial virus (RSV) causes most of the severe LRTIs in young children [7], while influenza viruses (IFVs) and human rhinoviruses (HRVs) are the predominant causative agents in hospitalized adults with pneumonia [8].

Shortly after the SARS events in 2002 [9], regional and nationwide laboratory-based surveillance studies for ARIs were conducted in China to help clarify the epidemiological feature of RVs by employing the highly sensitive modern molecular techniques, such as polymerase chain reaction (PCR) [10–16]. These studies are very helpful for characterizing the prevalence and burden of specific RVs in pre-vaccine Chinese populations (i.e., vaccines against *Streptococcus pneumoniae*, *Haemophilus influenzae* type b, and influenza viruses are currently not incorporated into the national immunization program [17]). In addition, these studies are useful to identify new emerging viral infections [18–21]. However, few previous studies have studied RVs across different hospital settings (e.g., outpatient, emergency room, inpatient wards and intensive care units) and explored etiological, environmental and host factors that predisposed patients to severe disease presentation, such as hospitalization or intensive care admission. It is very important to clarify the most common viral agents causing ARIs and their relationship with the severe disease presentation for clinicians who treated their patients with ARIs at different hospital settings.

The purpose of the study was to determine the prevalence of RVs in patients with ARIs at different hospital settings and to identify factors that were most likely associated with hospitalization due to ARIs.

## Methods

### Patient enrollment

The patients were enrolled from January 1, 2012 through December 31, 2015 at 11 hospitals (Additional file 1) in North China according to the following criteria: (1) had symptoms of acute infection, defined as fever (a body temperature > 38.0 °C) or hypothermia (a body temperature < 35.5 °C), chill, or leukocytosis (a white blood cell count > 10,000/ml) or leukopenia (a white blood cell count < 4000/ml); (2) had signs/symptoms of acute respiratory illness, defined as sore throat, runny nose, cough, sputum-production, shortness of breath, wheezing or crackles, or chest pain; and (3) with or without radiograph abnormality.

Patients were screened for enrollment eligibility by attending physicians of the 11 hospitals in the following settings: emergency department (ED), outpatient (internal medicine, pediatric, respiratory or febrile illness clinics) and/or inpatient (the respiratory medicine ward or the intensive care unit). The first two to five eligible patients (children and adults) in each hospital were enrolled each week via convenience sampling. Enrollment was conducted simultaneously in both inpatient and outpatient/ED settings in four hospitals. Five hospitals enrolled patients in outpatient/ED settings and two hospitals enrolled patients in inpatient settings. To avoid duplicated inclusion, patients who were referred from other hospitals or not initially diagnosed in our 11 hospitals were excluded. The study is observational and did not intervene with the choice of clinical management, namely hospitalization or ambulatory care.

### Data and specimen collection

Respiratory specimens (nasopharyngeal swab or aspirate, and sputum), regardless of the clinical severity, were regularly collected by physicians as quick as possible after patient enrollment. Bronchoalveolar lavage or pleural puncture fluid were collected upon physicians’ orders. The collected respiratory specimens were stored immediately in viral transportation media (VTM, Copan, Brescia, Italy) at − 70 °C at the hospitals before being transported to the central laboratory for viral screening. Demographic and clinical information were collected from each enrolled patient via a standardized case reporting form.

### Laboratory testing

Total nucleic acids (DNA and RNA) were extracted from respiratory specimens using RNA/DNA mini-kits (Qiagen, Valencia, CA) or Nucliens easMAG (bioMérieux, Marcy I′ Etoile, France), according to the manufacturers’ instructions. The presence of IFVs (A, B and C), HRVs, parainfluenza viruses (PIVs 1–4), RSV (A and B), human adenoviruses (HAdVs), human bocaviruses (HBoV), human metapneumovirus (hMPV), and human coronaviruses (HCoV-229E, OC43, NL63 and HKU1) were screened by reverse-transcriptase polymerase chain reaction (RT-PCR) and PCR assays as described previously [10].

### Statistical analysis

The prevalence (or detection rate) of viruses was calculated by dividing the number of positive cases by the total case numbers tested for that virus. The 95% binomial confidence interval (95% CI) for detection rates were calculated by the Pearson-Klopper method. To compare variables between subgroups of patients, we used Chi-square tests or Fisher’s exact tests for categorical variables and Wilcoxon rank-sum or Kruskal–Wallis tests for continuous variables as appropriate. Two-sided *p* value of < 0.05 was considered statistically significant.

To explore factors that predispose patients to hospitalization, multivariable logistic-regression modeling was used. The dependent variable was hospitalization (hospitalized =1, outpatient/ED patient = 0). The independent variables were the presence or absence of specific viral pathogen (identified = 1, unidentified = 0). To assess and quantify the contribution of a specific virus without regard to the presence of other pathogens, this model includes all tested respiratory viruses as well as their two-way interactions. In this case, the effect of co-infection upon hospitalization of ARIs, assigned as interaction term of pairs of viruses, could be explained as the excessive increment/decrement of hospitalization that could not be explained by additive effects or confounding of either composite virus. Variables (e.g., age, gender, surveillance year, and season of illness onset), were also included in multivariable model to account for potential confounding if significant at *p* < 0.10 in bivariate analysis. Since we hypothesized that the prevalence of RVs was disproportionately affected by the age of patients, multivariable models were constructed for patients of all ages, children aged 0–14 years, adults aged 15–64 years and elderly aged ≥65 years. The results of multivariable analyses were exhibited as adjusted odds ratio (aOR), namely the exponential of model coefficients. As we included a large number of cross-product terms in our model, type I errors and over-fitting will be significant problems. We used 99% CI for aORs instead in the multivariable analyses. All statistical analyses were conducted using R version 2.15.3 (R Foundation for Statistical Computing, Vienna, Austria) [22].

## Results

### Demographic and clinical characteristics

In total, 9924 patients were enrolled from the 11 hospitals. Of these patients, 3487 (35.1%) were hospitalized cases and 6437 (64.9%) were outpatient/ED patients. The median age of hospitalized cases was 8.8 years (interquartile range [IQR]: 3.0–52.0 years), which was much lower than the median age of outpatient/ED patients (28.2 years, IQR: 15.6–42.1 years) (*p* < 0.001). Compared with outpatient/ED patients, the hospitalized cases were more frequently male (58.3% vs. 51.7%, p < 0.001), children less than five years old (34.6% vs. 12.3%, p < 0.001) and elderly patients ≥65 years (17.3% vs. 6.3%, p < 0.001) (Table 1).

**Table 1:** Demographic and clinical characteristics of enrolled patients with ARIs in North China, 2012–2015

Characteristics	All patients (*n* = 9924), no. (%)	Hospital settings		
		Outpatient/ED, (*n* = 6437), no. (%)	Hospitalized, (*n* = 3487), no. (%)	*P*-value
Male	5362 (54.0)	3329 (51.7)	2033 (58.3)	< 0.001
Age, median years (IQR)	25.5 (6.6–44.3)	28.2 (15.6–42.1)	8.8 (3.0–52.0)	< 0.001*
Age in years				< 0.001
0–1	1014 (10.2)	272 (4.2)	742 (21.3)	
2–4	983 (9.9)	519 (8.1)	464 (13.3)	
5–14	1610 (16.2)	760 (11.8)	850 (24.4)	
15–34	3070 (30.9)	2746 (42.7)	324 (9.3)	
35–64	2240 (22.6)	1735 (27.0)	505 (14.5)	
≥ 65	1007 (10.1)	405 (6.3)	602 (17.3)	
Surveillance year				< 0.001
2012	2517 (25.4)	1804 (28.0)	713 (20.4)	
2013	2288 (23.1)	1201 (18.7)	1087 (31.2)	
2014	3198 (32.2)	2100 (32.6)	1098 (31.5)	
2015	1921 (19.4)	1332 (20.7)	589 (16.9)	
Season of illness onset ^a^				< 0.001
winter	2810 (28.3)	1510 (23.5)	712 (20.4)	
spring	2462 (24.8)	1789 (27.8)	1021 (29.3)	
summer	2222 (22.4)	1528 (23.7)	934 (26.8)	
autumn	2430 (24.5)	1610 (25.0)	820 (23.5)	
Clinical diagnosis				< 0.001
URTIs ^b^	5230 (52.7)	5069 (78.7)	161 (4.6)	
LRTIs ^c^	4694 (47.3)	1368 (21.3)	3326 (95.4)	
Pneumonia ^d^	4208 (42.4)	1134 (17.6)	3074 (88.2)	
Other LRTIs	486 (4.9)	234 (3.6)	252 (7.2)	

*. Wilcoxon test

^a^. spring = March to May; summer = June to August; autumn = September to November; winter = December to February

^b^. URTIs = upper respiratory tract infections, classified when common cold, rhinitis, pharyngitis, laryngitis or otitis media were diagnosed by attending physicians

^c^. LRTIs = lower respiratory tract infections, classified when pneumonia, bronchiolitis, bronchitis or exacerbations of chronic obstructive pulmonary disease and asthma were diagnosed

^d^. Pneumonia: chest X-ray showing evidence of consolidation (a dense or fluffy opacity with or without air bronchograms), other infiltrate (linear and patchy alveolar or interstitial densities), or pleural effusion

Of all patients, 5230 (52.7%) were URTIs and 4694 (47.3%) were LRTIs. Compared with outpatient/ED patients, the hospitalized cases were diagnosed more often with LRTIs (95.4% vs. 21.3%, *p* < 0.001) and pneumonia (88.2% vs. 17.6%, p < 0.001), but less often with URTIs (4.6% vs. 78.7%, *p* < 0.001). Moreover, some of clinical sign/symptoms were more frequent among the hospitalized cases than the outpatient/ED patients, including cough, wheezing/crackles, tachypnea, dyspnea, anemia, and leukocytosis (p < 0.001 for each symptom). In contrast, fever of > 38.5 °C, malaise, headache, rhinorrhea, and sore throat (p < 0.001 for each symptom) were more frequent among the outpatient/ED patients.

### Prevalence of viruses

**Fig. 1:**
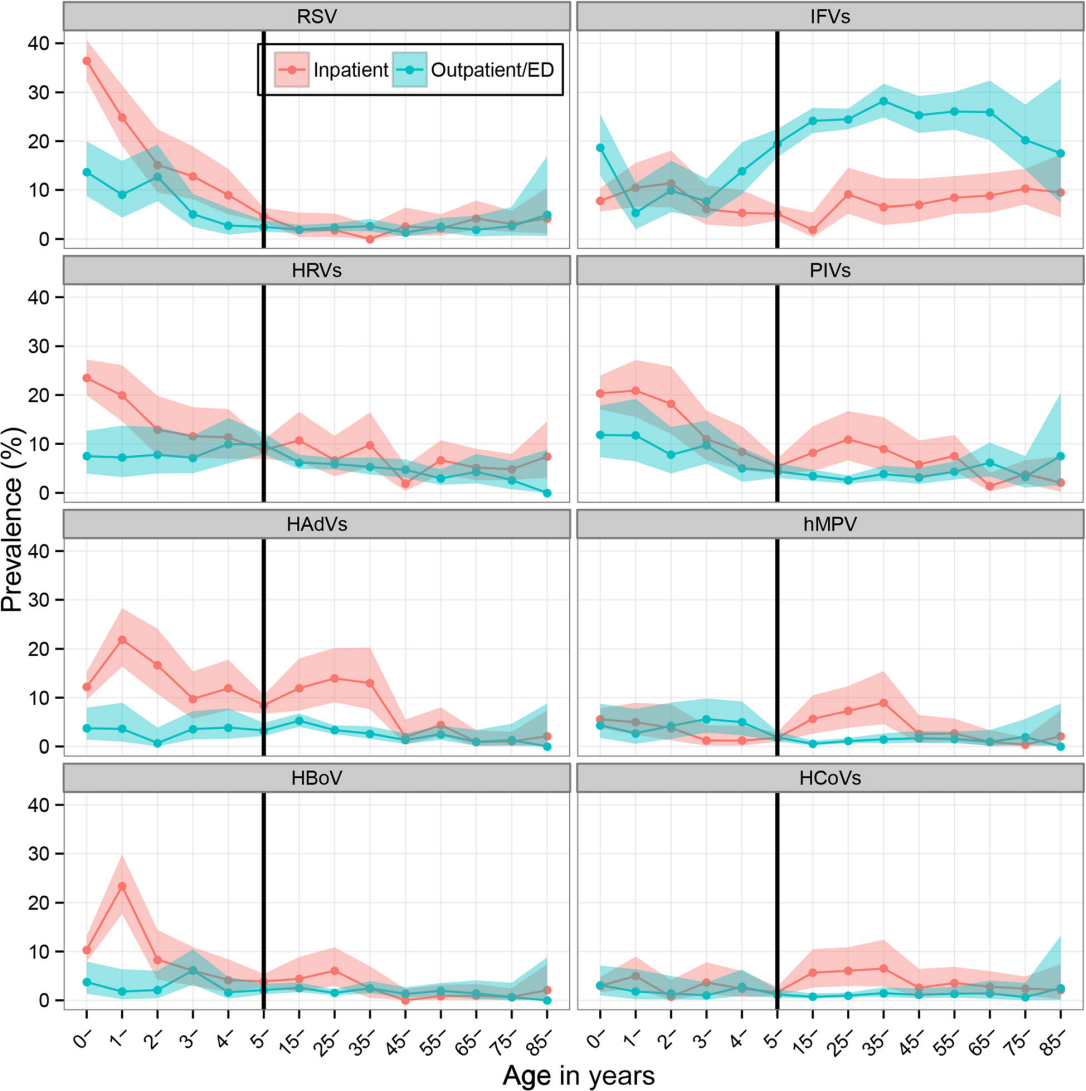
Frequency of respiratory viruses among patients with ARIs in North China, 2012–2015, by age group and hospital settings. The colored ribbons show 95% binomial confidence intervals of detection rates. Abbreviation: IFVs, Influenza viruses; HRVs, Human rhinoviruses; PIVs, Human parainfluenza viruses; RSV, Respiratory syncytial virus; HAdVs, Human adenoviruses; HBoV, Human bocaviruses; hMPV, Human metapneumovirus; HCoVs, Human coronaviruses

At least one virus was detected in 1369 (39.3%) hospitalized cases and 2459 (38.2%) outpatient/ED patients (*p* = 0.31). The overall detection rates of RVs varied disproportionately according to age of patients and hospital settings. The highest rates of RV infection were in children under two years of age (72.6% and 47.1% in hospitalized and outpatient/ED patients, respectively; p < 0.001) and the lowest rates of RV infection were in patients ≥65 years (22.9% and 35.1% in hospitalized and outpatient/ED patients, respectively; p < 0.001) (Table 2). The prevalence of specific RVs was significantly higher (*p* < 0.05) in hospitalized patients compared to outpatient/ED patients for specific ages. For instance, RSV, HRVs, HAdVs, PIVs and HBoV in young children and HAdVs, PIVs, hMPV and HCoVs in young adults were more frequent in the hospitalized patients. The exception was IFVs, which was more frequent in outpatient/ED patients (Fig. 1 & Table 3). Among the hospitalized patients, the viruses detected most frequently were RSV (33.3%, *n* = 303/1206) in children under two years, HAdVs (13.0%, *n* = 42/324) in patients 15–34 years, and IFVs (9.6%, *n* = 58/602) in elderly patients ≥65 years. However, among outpatient/ED patients the most common virus detected was IFVs across all age groups (22.7%, *n* = 1463/6437).

**Table 2:** Frequency of respiratory viruses single infection and co-infection among patients with ARIs in North China, 2012–2015, by age group and hospital settings

Age in years	Single virus infection (*n* = 3072)		*P*-value	Co-infection of viruses (*n* = 756)		*P*-value	Total (*n* = 3828)		*P*-value
	Outpatient/ED, No. (%)	Hospitalized, No. (%)		Outpatient/ED, No. (%)	Hospitalized, No. (%)		Outpatient/ED, No. (%)	Hospitalized, No. (%)	
0–1	105 (38.6)	278 (37.5)	0.77	23 (8.5)	261 (35.2)	< 0.001	128 (47.1)	539 (72.6)	< 0.001
2–4	179 (34.5)	148 (31.9)	0.416	27 (5.2)	70 (15.1)	< 0.001	206 (39.7)	218 (47.0)	0.024
5–14	245 (32.2)	209 (24.6)	< 0.001	44 (5.8)	53 (6.2)	0.753	289 (38.0)	262 (30.8)	0.003
15–34	896 (32.6)	66 (20.4)	< 0.001	123 (4.5)	31 (9.6)	< 0.001	1019 (37.1)	97 (29.9)	0.012
35–64	604 (34.8)	85 (16.8)	< 0.001	71 (4.1)	30 (5.9)	0.088	675 (38.9)	115 (22.8)	< 0.001
65+	131 (32.3)	126 (20.9)	< 0.001	11 (2.7)	12 (2.0)	0.521	142 (35.1)	138 (22.9)	< 0.001
Total	2160 (33.6)	912 (26.2)	< 0.001	299 (4.6)	457 (13.1)	< 0.001	2459 (38.2)	1369 (39.3)	0.31

**Table 3:** Frequency of respiratory viruses among patients with ARIs in North China, 2012–2015, by age group and hospital settings

Respiratory Viruses	Outpatient/ED patients, No. (%)				Hospitalized patients, No. (%)			
	Age 0–14 y, (*N* = 1551)	Age 14–64 y (*N* = 4481)	Age 65+ y (*N* = 405)	All (N = 6437)	Age 0–14 y (*N* = 2056)	Age 14–64 y (*N* = 829)	Age 65+ y (*N* = 602)	All (N = 3487)
Respiratory syncytial virus	84 (5.4)	98 (2.2)	10 (2.5)	192 (3.0)	343 (16.7)	15 (1.8)	22 (3.7)	380 (10.9)
Influenza viruses	238 (15.3)	1132 (25.3)	93 (23.0)	1463 (22.7)	141 (6.9)	56 (6.8)	58 (9.6)	255 (7.3)
Human rhinoviruses	138 (8.9)	240 (5.4)	13 (3.2)	391 (6.1)	296 (14.4)	58 (7.0)	32 (5.3)	386 (11.1)
Parainfluenza viruses	104 (6.7)	146 (3.3)	21 (5.2)	271 (4.2)	253 (12.3)	68 (8.2)	16 (2.7)	337 (9.7)
Human adenoviruses	50 (3.2)	151 (3.4)	4 (1.0)	205 (3.2)	240 (11.7)	71 (8.6)	7 (1.2)	318 (9.1)
Human metapneumovirus	50 (3.2)	51 (1.1)	5 (1.2)	106 (1.6)	64 (3.1)	42 (5.1)	5 (0.8)	111 (3.2)
Human bocaviruses	42 (2.7)	87 (1.9)	4 (1.0)	133 (2.1)	164 (8.0)	22 (2.7)	6 (1.0)	192 (5.5)
Human coronaviruses	25 (1.6)	47 (1.0)	5 (1.2)	77 (1.2)	51 (2.5)	39 (4.7)	15 (2.5)	105 (3.0)

Viral co-infection was detected in 756 (7.6%) cases, of which 534 (70.6%) were dual-infection, 150 (19.8%) were triple-infection, and 72 (9.5%) were co-infected by more than four different RVs. Patients with more viruses detected were more likely to be hospitalized. Specifically, patients infected by one, two, three, or more than four types of RVs were hospitalized 29.7%, 56.0%, 68.0% and 77.8%, respectively (*p* < 0.001). The hospitalized cases generally had more co-infections than outpatient/ED patients (13.1% vs. 4.6%, p < 0.001) (Table 2). Among the hospitalized cases, RSV co-infected with HRVs (RSV:HRVs) was the most common combination identified in children less than five years old (5.5%, *n* = 66/1206) and HAdVs:PIVs in patients five years and older (1.7%, *n* = 39/2281). Among outpatient/ED patients, the most common combinations were IFVs:HRVs in children less than five years old (0.9%, *n* = 7/791), and HAdVs:HBoV in patients five years and older (1.2%, n = 66/5646) (Additional file 1).

### Risk factors for hospitalization

**Fig. 2:**
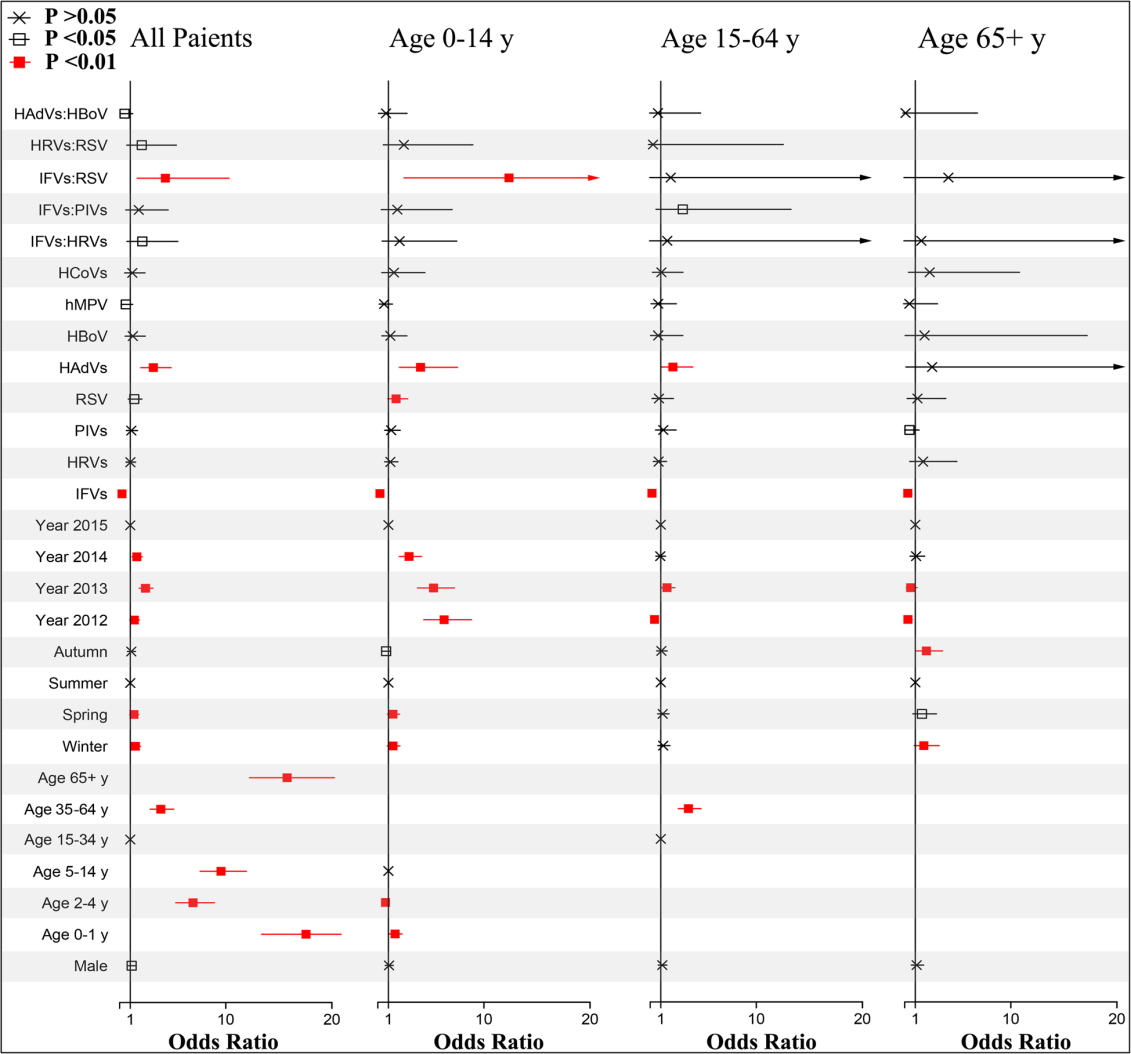
Adjusted Odds Ratios for risk factors or viruses associated with hospitalization of patients with ARIs, according to age groups. Horizontal lines indicate 99% confidence intervals. The reference group is assigned aOR = 1. Abbreviation: IFVs, Influenza viruses; HRVs, Human rhinoviruses; PIVs, Human parainfluenza viruses; RSV, Respiratory syncytial virus; HAdVs, Human adenoviruses; HBoV, Human bocaviruses; hMPV, Human metapneumovirus; HCoVs, Human coronaviruses

All viruses were associated with hospitalization in bivariate analyses (p < 0.001) (Additional file 1). However, multivariable analyses revealed that only 3 viruses were consistently associated with hospitalization after adjustment for other viruses and covariates. HAdVs (aOR: 4.03, 99% CI: 2.21–7.34) and RSV (aOR: 1.72, 99% CI: 1.11–2.66) as well as RSV co-infected with IFVs (aOR: 12.36, 99% CI: 2.63–58.03) were independently associated with increased hospitalization among children < 14 years. HAdVs were also associated with hospitalization among adults 15–64 years old (aOR: 2.14, 99% CI: 1.19–3.85). In contrast, infection of IFVs (aOR: 0.22, 99% CI: 0.17–0.28) was strongly associated with outpatient/ED patients in all age groups (Fig. 2).

Age was a strong host predicator of hospitalization. Young children less than two years (aOR: 17.56, 99% CI: 13.54–22.78) and elderly patients ≥65 years (aOR: 15.78, 99% CI: 12.41–20.07) had the highest risk compared with adults aged 15–34 years. The time of year also revealed environmental risk factors for these groups. For instance, compared to summer, children < 14 years who were infected during winter (aOR: 1.43, 99% CI: 1.06–1.91) and spring (aOR: 1.42, 99% CI: 1.08–1.89) were more frequently associated with hospitalization. However, elderly patients who infected during winter (aOR: 1.81, 99% CI: 1.06–3.08) and autumn (aOR: 2.05, 99% CI: 1.23–3.39) were more frequently associated with hospitalization (Fig. 2).

## Discussion

Early acquisition of viral diagnosis is helpful for clinicians when treating patients with ARIs by decreasing unnecessary prescription of antibiotics, guiding appropriate use of antiviral agents, and preventing virus transmission in both outpatient and inpatient settings [23]. The wide use of modern molecular techniques enables rapid diagnosis and greatly improves our understanding on the clinical role of respiratory viruses [24]. The high sensitivity of these testing methods reveals multiple pathogens in respiratory specimens (about 12%–42% of hospitalized children with ARIs had two or more viruses identified [5, 6, 25]). However, these results were often clinically confusing as it is difficult to distinguish whether the viruses were colonized or prolonged shedding from infection [26]. A better understanding of the distribution of a specific RVs in patients with ARIs in different hospital settings and their interactive relationship with other microbial agents can help clinicians make a better assessment of the role of RVs in the current infections. Toward this end, we simultaneously screened for the presence of eight common respiratory viruses in 9924 patients with ARIs in outpatient and inpatient settings at 11 hospitals in North China. After adjusting for other viruses and significant confounding factors, the detection of RSV, HAdVs, and co-infection of RSV with IFVs was still significantly higher in hospitalized children (or HAdVs in adults) with ARIs than in non-hospitalized children. These data suggest that these infections might predict the severity of ARIs.

RSV is a major viral agent identified in severe LRTIs in young children [27]. In our study, 95.4% of hospitalized cases were diagnosed with LRTIs, of which one third (34%) were young children. Of these hospitalized young children, one third (33%) had RSV detected. These results are highly consistent with previous studies, in which RSV infections were implicated in about 31%–39% of hospitalized children [4, 5, 28–30]. In addition, in our study RSV was found more frequently among hospitalized young children than outpatients and was a significant predicator of hospitalization among young children with ARIs. This pattern is consistent with that of previous studies [5, 28]. Our results confirmed that RSV was still an important health threat to pediatric populations in North China. These data highlight the need to develop effective vaccines and new therapeutics to better treat RSV in North China [31].

IFVs are important causing agents of ARIs [4, 30], and were reported to be the most common identified agent in hospitalized adults [8, 32, 33]. In our study, IFVs was also the most common agent in hospitalized patient ≥65 years. Interestingly, co-infection of RSV with IFVs was a significant predicator of increased hospitalization among young children in our study. Although IFVs were found more frequently among outpatient/ED patients (22.7%) than among hospitalized cases (7.3%), 91.1% of children co-infected with RSV and IFVs were admitted to the hospital. This was significantly higher than children hospitalized with the single infection of RSV (72.2%, *n* = 166/230) or IFVs (23.8%, *n* = 57/240). Based on these observations, we hypothesized that co-infection of IFVs with other microbial agents, like RSV in our study, might have played an important role in the hospitalization of ARIs in young children as has been described previously [34]. Both RSV and IFVs have caused substantial burden worldwide [7, 35, 36], and the epidemic season of RSV and IFVs are partly overlapping for winter months in China [11]. If our hypothesis that co-infection of IFVs with other microbial agents will cause more severe ARIs among young children is true, the immunization of young children with seasonal influenza vaccine would certainly offer additional benefits by preventing not only IFVs-associated hospitalizations, but also the excessive hospitalizations caused by co-infected RSV or other microbial agents. Since currently no influenza vaccines have been introduced into China’s routine immunization program, vaccination of the elderly people and young children with seasonal influenza vaccine should be considered a high priority to reduce the large burden of ARIs among these population groups at high risk of more severe disease.

In our study, viral infection was more common in inpatients than outpatients among younger age group but not older age group. This observation suggests that RVs infection is common among young children and is more likely to be associated with severe ARIs when compared with other age groups, while in adults and elderly people, though RVs infection is not uncommon, RVs are less likely to cause severe ARIs in this age group. Moreover, we also found co-infection of two or more viruses in 15%–35% of hospitalized young children as opposed to 2%–10% of hospitalized adults. Higher proportions of co-infection were observed in hospitalized young children than in non-hospitalized children (5%–9%). These results suggest that viral co-infection might predict hospitalization of young children but not of adults. Aging is a protecting factor for RVs infections in young children. As the age of children increased, infection and co-infection of RVs became less frequent and were less likely to be associated with hospitalization. This observation suggested that the frequent exposure of children to RVs could lower their risk of RVs-associated infection and/or severe infection, and that the utility of vaccines, when available, could be used to control and prevent the infection of RVs. The most frequent combination of viral co-infection among hospitalized children was RSV with HRVs, which is consistent with that of previous studies [5, 6, 25, 37, 38]. In studies by Papadopoulos et al. and Aberle et al., co-infection of RSV with HRVs was associated with prolonged stay in the hospital in patients with bronchiolitis [25, 37]. In our study, however, the strength of association with hospitalization for RSV co-infected with HRVs was at a marginal value (significant at *p* < 0.05 but not *p* < 0.01), which warrants investigation in future studies.

In our study, HAdVs were the most common viral agents among adolescents and young adults hospitalized with ARIs, which is consistent with previous studies [33, 39]. In addition, HAdVs in our study were more frequent in hospitalized children and young adults than in outpatient/ED patients. HAdVs are important agents associated with severe ARIs, for several serotypes of HAdVs (e.g., HAdV-3, 7, 14 and 55), might induce severe and fatal necrotizing pneumonia [26, 40–42]. The host response to HAdVs infection is very similar to that of invasive bacterial infections [43]. In our previous study of the same population in North China, we found that 49% of identified HAdVs were HAdV-7 [44]. We believe that certain serotypes of HAdVs, e.g. HAdV-7, were more prevalent in this population, which played an important role in the hospitalization of children and young adults in our study. Future studies which sought to sequence the identified HAdVs strains are warranted.

Our study had limitations. First, we did not include an asymptomatic control in our study but rather made comparisons between patient subgroups at different hospital settings which might make the results difficult to interpret. Yet finding no difference does not mean that there is no association of specific virus with the hospitalization of patients. However, the heterogeneous distribution of RVs in patient subgroups at different hospital settings deserved attention from clinicians when treating these patients. Second, we only investigated 2-way interactions of viruses. The interactions and bacterial-viral infections were not explored. Third, other host factors were not examined in our study, such as those underlying diseases (e.g. exacerbation of chronic obstructive pulmonary disease, asthma, or cardiovascular diseases), and the previous usage of antibiotics or steroids. The issue of viral-viral co-infection associated with severity of ARIs still remains controversial as laid down by others [6, 45, 46]. Future studies that test multi-pathogens using common standards at multi-sites, employ appropriate community controls and use death as clinical endpoints are warranted.

## Conclusions

In conclusion, a substantial proportion of ARIs are associated with the infection or co-infection of RVs in North China. RSV, HAdVs, and co-infection of RSV and IFVs were more frequent in hospitalized children (or adenoviruses in adults), which might predict the severity of ARIs. Thus, clinicians treating patients with ARIs should be vigilant of these infections. Our findings provide valuable insights into the study of viral pathogenesis and provide guidance for good clinical management of severe ARIs.

## Additional file

Additional file 1 **Table S1.** Hospitals that participated in the study of acute respiratory infections in North China, 2012–2015. **Table S2.** Respiratory viruses detected in patients with acute respiratory infections in North China, 2012–2015, by age group and hospital setting. **Figure S1.** Frequency of co-infected viruses in patients with ARIs in North China, 2012–2015, by age group and hospital settings. (DOCX 391 kb)

## Abbreviations

95% CI: 95% confidence interval
aOR: adjusted odds ratio
ARIs: Acute respiratory infections
ED: Emergency department
HAdVs: Human adenoviruses
HBoV: Human bocaviruses
HCoVs: Human coronaviruses
hMPV: human metapneumovirus
HRVs: Human rhinoviruses
IFVs: Influenza viruses
LRTIs: Lower respiratory tract infections
PIVs: Parainfluenza viruses
RSV: Respiratory syncytial virus
RT-PCR: Reverse-transcriptase polymerase chain reaction
RVs: Respiratory viruses
URTIs: Upper respiratory tract infections
