# Supplementary material for: Comparison of the prevalence of respiratory viruses in patients with acute respiratory infections at different hospital settings in North China, 2012–2015
Source: BMC Infect Dis. 2018 Feb 8;18:72. doi: 10.1186/s12879-018-2982-3 (PMC5806372; doi:10.1186/s12879-018-2982-3)
Supplement: Supplementary file 1 — Table S1. Hospitals that participated in the study of acute respiratory infections in North China, 2012–2015. Table S2. Respiratory viruses detected in patients with acute respiratory infections in North China, 2012–2015, by age group and hospital setting. Figure S1. Frequency of co-infected viruses in patients with ARIs in North China, 2012–2015, by age group and hospital settings. (DOCX 391 kb) [file 12879_2018_2982_MOESM1_ESM.docx]

**Table S1.** Hospitals that participated in the study of acute respiratory infections in North China, 2012-2015

| ID | Province | Hospital | Hospital settings |
| --- | --- | --- | --- |
| 1 | Beijing | Peking Union Medical College Hospital | Inpatient |
| 2 | Beijing | Peking University People's Hospital | Both |
| 3 | Beijing | Beijing Children's Hospital | Both |
| 4 | Beijing | Peking University Sixth Hospital | Outpatient/ED |
| 5 | Beijing | Beijing Dongfang Hospital | Outpatient/ED |
| 6 | Beijing | Beijing Chuiyangliu Hospital | Outpatient/ED |
| 7 | Beijing | Beijing Longfu Hospital | Outpatient/ED |
| 8 | Beijing | Beijing Luhe Hospital | Both |
| 9 | Beijing | Beijing Shunyi Distrit Hospital | Both |
| 10 | Shandong | Jinan Central Hospital | Outpatient/ED |
| 11 | Shandong | Qilu Children's Hospital of Shandong University | Inpatient |

**Table S2.** Respiratory viruses detected in patients with acute respiratory infections in North China, 2012-2015, by age group and hospital setting

| Age in years | Respiratory syncytial virus  (n=572) | | P-value | Influenza viruses  (n=1718) | | P-value | Human rhinoviruses  (n=777) | | P-value | Human adenoviruses  (n=523) | | P-value |
| --- | --- | --- | --- | --- | --- | --- | --- | --- | --- | --- | --- | --- |
|  | Outpatient/ED No. (%) | Hospitalized,  No. (%) |  | Outpatient/ED No. (%) | Hospitalized,  No. (%) |  | Outpatient/ED No. (%) | Hospitalized,  No. (%) |  | Outpatient/ED No. (%) | Hospitalized,  No. (%) |  |
| <1 | 22(13.7) | 197(36.4) | <0.001 | 30(18.6) | 42(7.8) | <0.001 | 12(7.5) | 127(23.5) | <0.001 | 6(3.7) | 66(12.2) | 0.001 |
| 1 | 10(9.0) | 50(24.9) | 0.001 | 6(5.4) | 21(10.4) | 0.146 | 8(7.2) | 40(19.9) | 0.003 | 4(3.6) | 44(21.9) | <0.001 |
| 2 | 18(12.7) | 20(15.2) | 0.602 | 14(9.9) | 15(11.4) | 0.699 | 11(7.7) | 17(12.9) | 0.169 | 1(0.7) | 22(16.7) | <0.001 |
| 3 | 10(5.1) | 21(12.8) | 0.013 | 15(7.7) | 10(6.1) | 0.679 | 14(7.1) | 19(11.6) | 0.199 | 7(3.6) | 16(9.8) | 0.028 |
| 4 | 5(2.8) | 15(8.9) | 0.019 | 25(13.8) | 9(5.4) | 0.011 | 18(9.9) | 19(11.3) | 0.73 | 7(3.9) | 20(11.9) | 0.008 |
| 5-14 | 19(2.5) | 40(4.7) | 0.023 | 148(19.5) | 44(5.2) | <0.001 | 75(9.9) | 74(8.7) | 0.439 | 25(3.3) | 72(8.5) | <0.001 |
| 15-24 | 21(1.9) | 3(1.9) | 1 | 266(24.2) | 3(1.9) | <0.001 | 68(6.2) | 17(10.7) | 0.042 | 58(5.3) | 19(11.9) | 0.002 |
| 25-34 | 39(2.4) | 3(1.8) | 1 | 403(24.5) | 15(9.1) | <0.001 | 96(5.8) | 11(6.7) | 0.606 | 55(3.3) | 23(13.9) | <0.001 |
| 35-44 | 18(2.6) | 0(0) | 0.092 | 193(28.2) | 8(6.5) | <0.001 | 36(5.3) | 12(9.8) | 0.062 | 18(2.6) | 16(13.0) | <0.001 |
| 45-54 | 7(1.3) | 4(2.6) | 0.279 | 136(25.3) | 11(7.1) | <0.001 | 25(4.7) | 3(1.9) | 0.166 | 7(1.3) | 3(1.9) | 0.702 |
| 55-64 | 13(2.5) | 5(2.2) | 1 | 134(26.1) | 19(8.4) | <0.001 | 15(2.9) | 15(6.6) | 0.025 | 13(2.5) | 10(4.4) | 0.174 |
| 65-74 | 4(1.9) | 9(4.2) | 0.26 | 55(25.9) | 19(8.8) | <0.001 | 9(4.2) | 11(5.1) | 0.82 | 2(0.9) | 2(0.9) | 1 |
| 75-84 | 4(2.6) | 9(3.1) | 1 | 31(20.3) | 30(10.3) | 0.005 | 4(2.6) | 14(4.8) | 0.32 | 2(1.3) | 3(1.0) | 1 |
| 85+ | 2(5.0) | 4(4.2) | 1 | 7(17.5) | 9(9.5) | 0.243 | 0(0) | 7(7.4) | 0.104 | 0(0) | 2(2.1) | 1 |
| Total | 192(3.0) | 380(10.9) | <0.001 | 1463(22.7) | 255(7.3) | <0.001 | 391(6.1) | 386(11.1) | <0.001 | 205(3.2) | 318(9.1) | <0.001 |

**Table S2.** Respiratory viruses detected in patients with acute respiratory infections in North China, 2012-2015, by age group and hospital setting (Continued)

| Age in years | Parainfluenza viruses  (n=608) | | P-value | Human metapneumovirus  (n=217) | | P-value | Human bocaviruses  (n=325) | | P-value | Human coronaviruses  (n=182) | | P-value |
| --- | --- | --- | --- | --- | --- | --- | --- | --- | --- | --- | --- | --- |
|  | Outpatient/ED No. (%) | Hospitalized,  No. (%) |  | Outpatient/ED No. (%) | Hospitalized,  No. (%) |  | Outpatient/ED No. (%) | Hospitalized,  No. (%) |  | Outpatient/ED No. (%) | Hospitalized,  No. (%) |  |
| <1 | 19(11.8) | 110(20.3) | 0.015 | 7(4.3) | 30(5.5) | 0.689 | 6(3.7) | 56(10.4) | 0.007 | 5(3.1) | 16(3.0) | 1 |
| 1 | 13(11.7) | 42(20.9) | 0.044 | 3(2.7) | 10(5.0) | 0.393 | 2(1.8) | 47(23.4) | <0.001 | 2(1.8) | 10(5.0) | 0.224 |
| 2 | 11(7.7) | 24(18.2) | 0.011 | 6(4.2) | 5(3.8) | 1 | 3(2.1) | 11(8.3) | 0.026 | 2(1.4) | 1(0.8) | 1 |
| 3 | 19(9.7) | 18(11.0) | 0.73 | 11(5.6) | 2(1.2) | 0.043 | 12(6.1) | 10(6.1) | 1 | 2(1.0) | 6(3.7) | 0.149 |
| 4 | 9(5.0) | 14(8.3) | 0.28 | 9(5.0) | 2(1.2) | 0.063 | 3(1.7) | 7(4.2) | 0.206 | 5(2.8) | 4(2.4) | 1 |
| 5-14 | 33(4.3) | 45(5.3) | 0.416 | 14(1.8) | 15(1.8) | 1 | 16(2.1) | 33(3.9) | 0.042 | 9(1.2) | 14(1.6) | 0.53 |
| 15-24 | 38(3.5) | 13(8.2) | 0.009 | 6(0.5) | 9(5.7) | <0.001 | 28(2.5) | 7(4.4) | 0.193 | 8(0.7) | 9(5.7) | <0.001 |
| 25-34 | 43(2.6) | 18(10.9) | <0.001 | 18(1.1) | 12(7.3) | <0.001 | 25(1.5) | 10(6.1) | 0.001 | 16(1.0) | 10(6.1) | <0.001 |
| 35-44 | 26(3.8) | 11(8.9) | 0.018 | 10(1.5) | 11(8.9) | <0.001 | 17(2.5) | 3(2.4) | 1 | 10(1.5) | 8(6.5) | 0.003 |
| 45-54 | 17(3.2) | 9(5.8) | 0.15 | 9(1.7) | 4(2.6) | 0.503 | 7(1.3) | 0(0) | 0.359 | 6(1.1) | 4(2.6) | 0.244 |
| 55-64 | 22(4.3) | 17(7.5) | 0.076 | 8(1.6) | 6(2.7) | 0.38 | 10(1.9) | 2(0.9) | 0.363 | 7(1.4) | 8(3.5) | 0.084 |
| 65-74 | 13(6.1) | 3(1.4) | 0.011 | 2(0.9) | 2(0.9) | 1 | 3(1.4) | 2(0.9) | 0.684 | 3(1.4) | 6(2.8) | 0.503 |
| 75-84 | 5(3.3) | 11(3.8) | 1 | 3(2.0) | 1(0.3) | 0.12 | 1(0.7) | 2(0.7) | 1 | 1(0.7) | 7(2.4) | 0.273 |
| 85+ | 3(7.5) | 2(2.1) | 0.154 | 0(0) | 2(2.1) | 1 | 0(0) | 2(2.1) | 1 | 1(2.5) | 2(2.1) | 1 |
| Total | 271(4.2) | 337(9.7) | <0.001 | 106(1.6) | 111(3.2) | <0.001 | 133(2.1) | 192(5.5) | <0.001 | 77(1.2) | 105(3.0) | <0.001 |


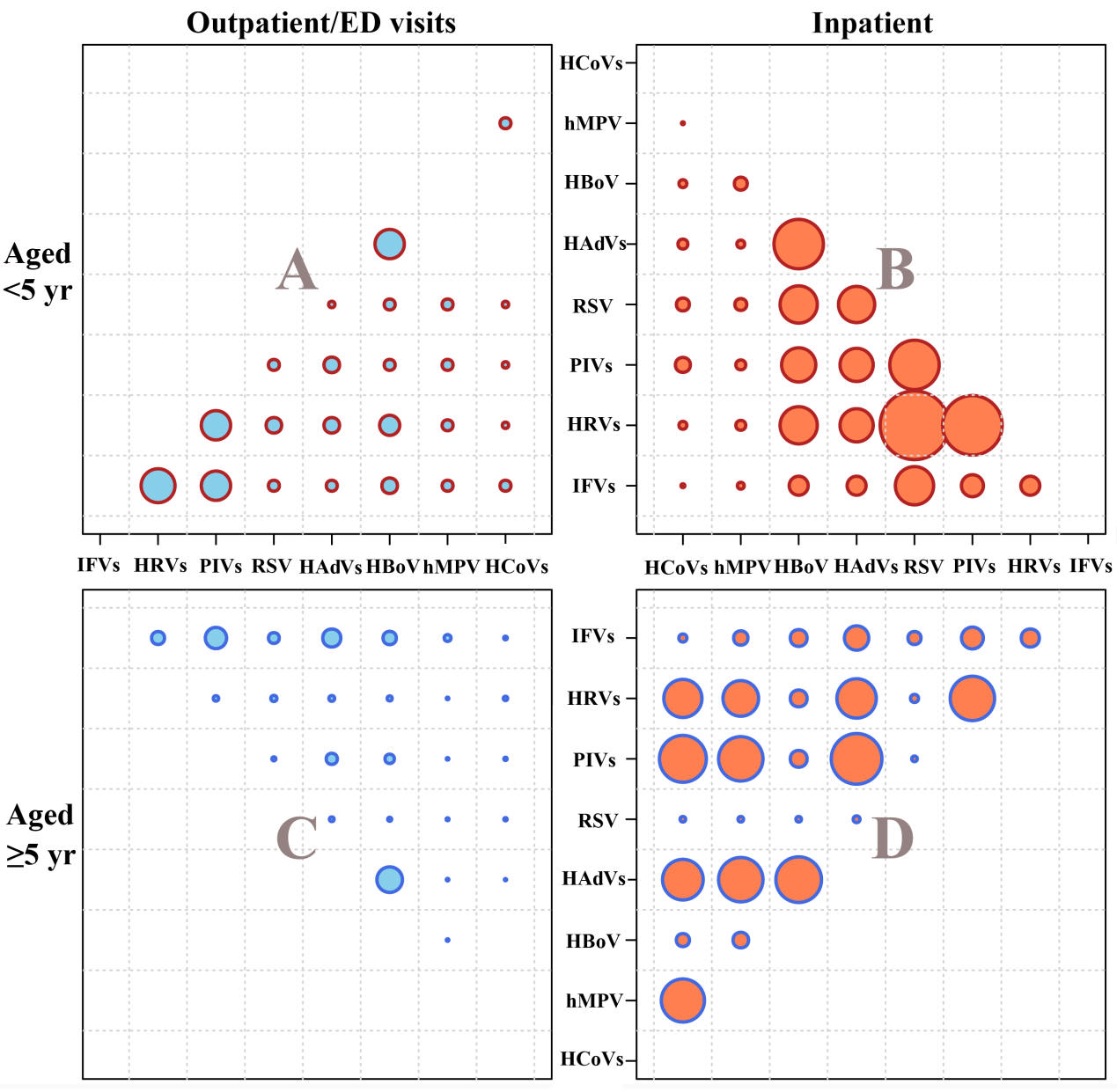


**Figure S1.** Frequency of co-infected viruses in patients with ARIs in North China, 2012-2015, by age group and hospital settings.

Panel (A): outpatient/ED patients younger than five years of age. Panel (B): hospitalized patients younger than five years of age. Panel (C): outpatient/ED patients five years and older. Panel (D): hospitalized patients five years and older.

The circle radius in the cells is proportionate to the prevalence of viruses co-infected.

Abbreviation: IFVs, Influenza viruses; HRVs, Human rhinoviruses; PIVs, Human parainfluenza viruses; RSV, Respiratory syncytial virus; HAdVs, Human adenoviruses; HBoV, Human bocaviruses; hMPV, Human metapneumovirus; HCoVs, Human coronaviruses.
